# A Dual-Band 8-Antenna Array Design for 5G/WiFi 5 Metal-Frame Smartphone Applications

**DOI:** 10.3390/mi15050584

**Published:** 2024-04-28

**Authors:** Huiyang Li, Shanshan Xiao, Lefei He, Qibo Cai, Gui Liu

**Affiliations:** College of Electrical and Electronic Engineering, Wenzhou University, Wenzhou 325035, China23451248008@stu.wzu.edu.cn (L.H.); caiqibo@wzu.edu.cn (Q.C.)

**Keywords:** dual-band antenna, MIMO antenna, metal frame, smartphone, 5G antenna

## Abstract

This paper presents a dual-band 8-port multiple-input multiple-output (MIMO) antenna specifically designed for fifth-generation (5G) smartphones, featuring two open-slot metal frames. To enhance impedance matching and improve isolation between adjacent antenna elements, each antenna element employed a coupling feed. All simulation results in this paper come from Ansys HFSS. The operational frequency bands of the proposed antenna spanned 3.36–4.2 GHz for the lower band and 4.37–5.95 GHz for the higher band, covering 5G New Radio (NR) bands N78 (3.4–3.6 GHz) and N79 (4.4–4.9 GHz), as well as WiFi 5 (5.15–5.85 GHz). Notably, the antenna demonstrated outstanding isolation exceeding 16.5 dB within the specified operating bands. The exceptional performance positions the proposed antenna as a promising candidate for integration into 5G metal-frame smartphones.

## 1. Introduction

The advent of the fifth-generation (5G) internet protocol has spurred a substantial demand for high-speed mobile data services. Achieving high transmission efficiency, low latency, and minimal energy consumption poses a significant challenge in current mobile communication systems. The 3rd Generation Partnership Project (3GPP) has identified three sub-6 GHz operating bands for 5G New Radio (NR): N77 (3.3–4.2 GHz), N78 (3.3–3.8 GHz), and N79 (4.4–5.0 GHz). In 2017, China allocated spectrum for 5G systems in sub-6 GHz, designating N78 (3.4–3.6 GHz) and N79 (4.8–4.9 GHz) as the operational frequency bands.

On one hand, multiple-input multiple-output (MIMO) antenna technology excels in enhancing system capacity and mitigating multipath interference effects. Various MIMO antennas operating in the sub-6 GHz spectrum have been documented [1,2,3,4,5,6,7,8,9]. Lower-order systems like 2 × 2 and 4 × 4 have gained popularity for fourth-generation (4G) smartphones in long-term evolution (LTE) bands [3]. The efficacy of massive MIMO antennas in achieving larger channel capacity and higher transmission rates for 5G systems is evident, garnering attention from both the industry and academia. Noteworthy MIMO antenna designs include folded monopoles and gap-coupled loop branches for dual-band operation [4], double-branch monopoles with a T-shaped decoupling stub for mutual coupling reduction [5], and an eight-port triple-band MIMO antenna for 5G metal-frame smartphones [6]. A multiband 10-antenna array for sub-6 GHz MIMO applications and a 10-port MIMO antenna with two types of antenna modules are presented in [7,8], respectively. An eighteen-antenna system compatible with a massive MIMO/Diversity 5G smartphone is proposed without external decoupling structures [9].

On the other hand, size constraints in multi-band MIMO systems often lead to strong mutual coupling effects between antenna elements. Decoupling methods such as decoupling branches, defected ground structures (DGS), neutralization lines (NL), parasitic elements, and metamaterials have been proposed to address these challenges. In this paper, a variety of defective ground structures are used for antenna design. The defected ground structures of the microstrip line are implemented by making an artificial defect on the ground and provide a resonance property in transfer characteristics [10]. This structure can change the distribution of the effective dielectric constant of the antenna dielectric substrate, thereby changing the distributed inductance and distributed capacitance of the microstrip line based on the medium, thereby giving such microstrip lines band gap characteristics and slow wave characteristics. According to the circuit theory [11], parallel RLC circuits work as a band-stop filter. By applying the same concept to defected ground structures in microstrip technology, the vertical slot of defected ground structures accumulates charge and increases the effective capacitor of the microstrip line. Two defected areas on both sides and one connecting slot correspond to the equivalently added inductance L and capacitance C, respectively. The defected ground structures can provide additional effective inductance of the transmission line. The increase in effective inductance from insertion of the defected ground structures can provide a longer electrical length of transmission line than that of a conventional line, which enables size reduction of the microwave and millimeter circuit. Compared with other structures, the defected ground structure has a simple structure; it has the advantages of simple production, small size, and easy integration, so it also has a certain competitiveness in the field of antenna applications [12].

Additionally, the metal frame is a distinctive feature of full-screen smartphones, gaining attention in recent years [13,14,15,16]. An antenna element encompassing C-shaped microstrip patches, two rectangular radiating microstrips on a metal frame, and a rectangular slot on the ground plane has been proposed, covering 5G N77 (3.3–4.2 GHz), N78 (3.3–3.8 GHz), N79 (4.4–5.0 GHz), and LTE46 (5.15–5.93 GHz) bands [13]. A metal-frame smartphone antenna design with dimensions of 150 mm × 70 mm × 6 mm incorporates an inverted-F antenna and an asymmetric T-slot on the circuit board edges, demonstrating significant engineering application value [16].

One of the primary challenges in contemporary mobile antenna design lies in ensuring optimal performance when the device is held by the user. With the escalating demand for broader frequency bands and the diminishing space allocated for antennas, achieving satisfactory performance, even in ideal free-space conditions, has become increasingly challenging [17]. In [18], an analysis of fundamental theories pertaining to models of objects (e.g., the human body) with high dielectric constants and significant losses offers a theoretical framework for subsequent hand model applications. Similarly, numerous case studies examining the interaction patterns between mobile phones and hands offer valuable insights into designing strategies aimed at enhancing the radiation characteristics of mobile phone antennas [19].

This paper introduces a 5G dual-band MIMO antenna operating at 3.36–4.2 GHz and 4.37–5.95 GHz frequencies. Eight elements are printed on two side-edge metal frames, and two types of decoupling structures (DS_1_ and DS_2_) are employed to mitigate mutual coupling. The proposed MIMO antenna undergoes manufacturing and testing, and measurement results, including S-parameters, radiation efficiencies, radiation patterns, and calculated envelope correlation coefficient (ECC), are discussed. The proposed antenna effectively covers the 5G China bands N78 (3.4–3.6 GHz) and N79 (4.4–5.0 GHz), along with WiFi 5 (5.15–5.85 GHz).

## 2. Proposed MIMO Antenna

### 2.1. Antenna Structure

**Figure 1:**
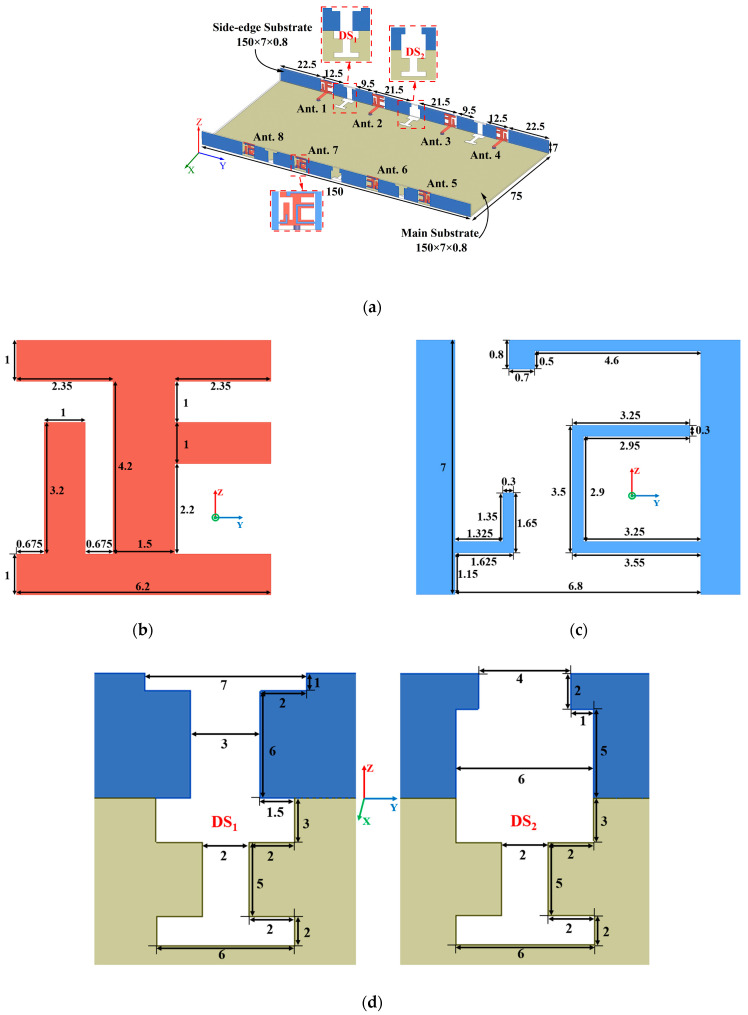

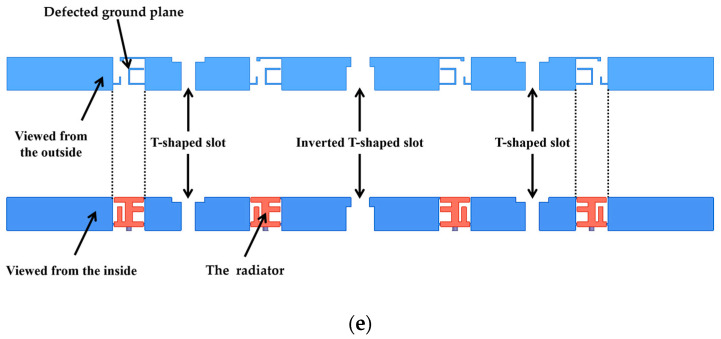
(**a**) Geometry of the proposed antenna array, (**b**) detailed structure of the radiator, (**c**) detailed structure of the defected ground plane, (**d**) detailed structure of the decoupling structures, (**e**) the relative position of various structures (all values are in millimeters).

The structure and physical dimensions of the proposed antenna are depicted in Figure 1a. The MIMO antenna design incorporates three FR-4 substrates, each exhibiting a relative permittivity of 4.4 and a loss tangent of 0.02. These substrates consist of a central main substrate and two additional side-edge substrates. The main substrate measures 150 mm × 73.4 mm × 0.8 mm, while the side-edge substrates have dimensions of 150 mm × 7 mm × 0.8 mm. Precisely printed on the two side-edge substrates are eight antenna elements, strategically positioned perpendicular to the main substrate.

### 2.2. Antenna Element

Each antenna element consists of a radiator, feeding line, and a defected ground plane. The radiator takes the form of a Chinese character 正-shaped strip, which consists of three horizontal rectangular microstrip lines and two longitudinal rectangular microstrip lines, precisely printed on the inner surface of the side-edge substrate. The defected ground plane is composed of a rectangular strip connected to an inverted L-shaped strip, and another rectangular strip attached to an inverted L-shaped strip and a C-shaped strip. The feeding line is printed on the top surface of the main substrate, establishing a connection with the radiator. Four antenna elements are printed on the inner surface of each side-edge substrate. To enhance isolation between these elements, two distinct decoupling structures (DS_1_ and DS_2_) are employed, as depicted in Figure 1d. Among them, the decoupling structure 1 consists of a T-shaped slot and an I-shaped slot, while the decoupling structure 2 is composed of an inverted T-shaped slot and an I-shaped slot. These structures are etched on the outer surface of the side-edge substrates and the bottom surface of the main substrate, effectively extending the coupling current path and minimizing interaction between different antenna elements. Viewed from different directions, the specific structure of the metal frame is as shown in Figure 1e, which shows the positional relationship of the structures mentioned above. Viewed from the inside of the antenna, the orthogonal structure of the 正-shaped strip is located on the inner surface of the side dielectric substrate, which can just cover the defected ground structure. These two structures are interconnected, jointly forming the radiating unit of the antenna during operation. In the figure, the defective ground plane of the antenna unit is located directly outside the 正-shaped strip. Looking from the outside of the mobile phone, the defective ground plane, T-shaped slot, and inverted T-shaped slot mentioned above are both located on the outer surface of the metal frame. Although the above two structures have the same principle, their distinct locations endow them with varied functions. The defected ground plane and the orthogonal structure constitute the antenna element, which is used to generate the resonant frequency at which the antenna operates, whereas the slots on the metal frame and the defective slots of the metal ground plane form a decoupling structure to enhance isolation between each antenna port.

**Figure 2:**
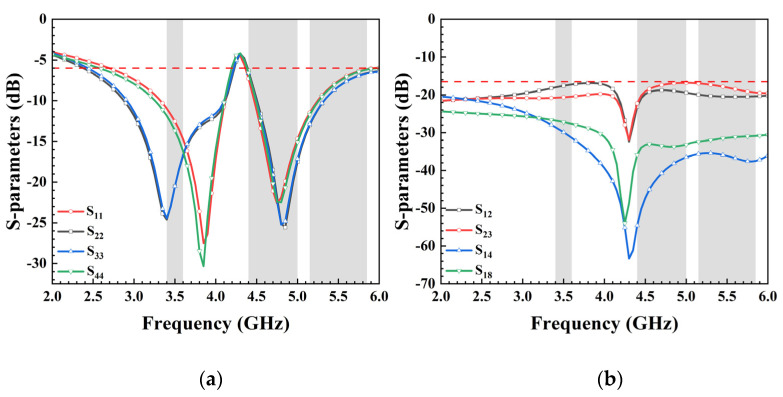
(**a**) Simulated reflection coefficients and (**b**) transmission coefficients of the proposed antenna.

Due to variations in the location and radiation environment of each antenna unit, the reflection coefficient of each unit differs slightly, despite their identical size. With the antenna elements sharing the same dimensions and structures and being symmetrically placed alongside decoupling structures, the eight units can be categorized into two groups: Ant. 1, Ant. 4, Ant. 5, and Ant. 8 form one category, while the remaining four antennas compose another. Therefore, only the reflection coefficients of Ant. 1, Ant. 2, Ant. 3, and Ant. 4 are given in Figure 2a. The antenna operates within the frequency range of 2.55–4.2 GHz and 4.4–6 GHz, effectively covering 5G China Bands N78 (3.4–3.6 GHz), N79 (4.4–5.0 GHz), and WiFi 5 (5.15–5.85 GHz). In this paper, S_18_ denotes the transmission coefficient between antenna elements 1 and 8, where S_18_ = S_45_, S_12_ = S_34_ = S_56_ = S_78_, S_23_ = S_67_, and S_14_ = S_58_. Figure 2b showcases the antenna’s isolation, revealing that all ports maintain isolation levels exceeding 16.5 dB, ensuring independence between antenna units and preventing mutual interference.

**Figure 3:**
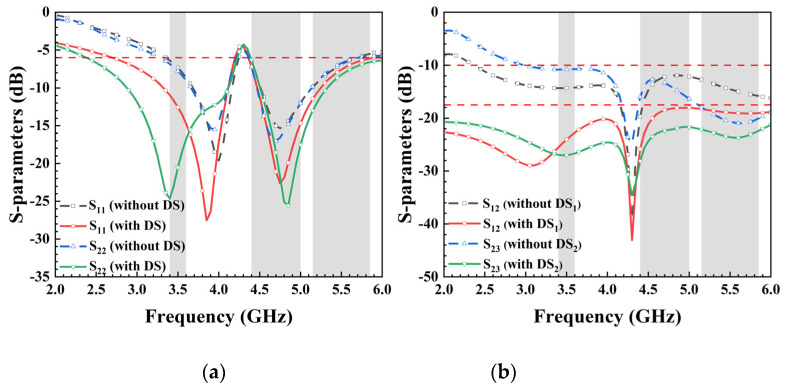
Comparison of simulated (**a**) reflection coefficients and (**b**) transmission coefficients between the antenna without decoupling structures and the proposed antenna.

In general, to enhance isolation and achieve low correlation, external decoupling structures are introduced between antenna elements. Designs with eight MIMO antennas often utilize an additional slot in the ground plane to improve isolation. As the equivalent circuit of this decoupling structure resembles a parallel RLC configuration, resonance will occur at specific frequency, consequently influencing the surface current distribution within the ground layer and altering the transmission line characteristics of the antenna unit. This study demonstrates that the slot not only enhances mutual isolation between antenna elements but also serves as the ground clearance area to expand bandwidth. Figure 3a presents simulation results of reflection coefficients with/without DS. The simulated S_11_ and S_22_ continue to cover desired bands even without DS. Figure 3b illustrates simulated isolation curves S_12_ and S_23_ with/without DS_1_ and DS_2_. The utilization of decoupling structures effectively attenuates mutual coupling at 3.5 GHz, 4.9 GHz, and 5.5 GHz. In the desired frequency bands, the low isolation (11 dB) at 3.5 GHz improves distinctly with the introduction of DS_1_ between Ant. 1 and Ant. 2. Furthermore, with the incorporation of DS_2_, the simulated S_23_ complies with the 15 dB requirement in the desired frequency bands.

## 3. Design Process

**Figure 4:**
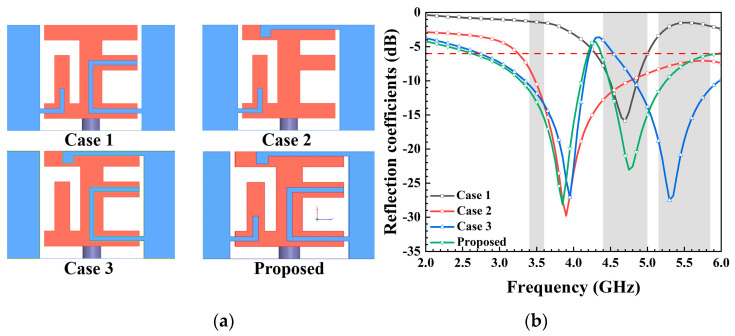
(**a**) Four cases of the antenna element during the evolution process, and (**b**) simulated reflection coefficients.

The design methodology for the antenna element is outlined in Figure 4a. Simulated reflection coefficients are presented in Figure 4b. The reflection coefficients for Case 1 and Case 2 indicate that the low-frequency resonance was generated by the inverted L-shaped strip atop the metal frame, while the high-frequency resonance was influenced by the C-shaped strip on the right side of the metal frame. Moreover, Case 3 demonstrates that the inverted L-shaped strip connected to the left rectangular strip effectively controlled the deviation between the two resonance points. Ultimately, the proposed antenna element covered the frequency range of 2.55–4.2 GHz in the lower band and 4.4–6 GHz in the higher band.

**Figure 5:**
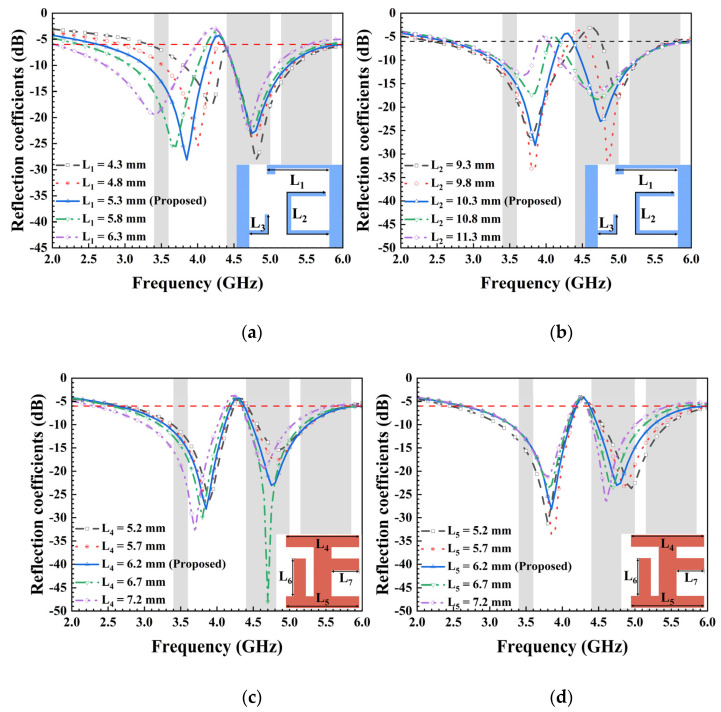
Simulated reflection coefficients with different values of (**a**) L_1_, (**b**) L_2_, (**c**) L_4_, and (**d**) L_5_.

Owing to disparities in radiation conditions and surface current distributions, key dimensions within the antenna yielded diverse effects on the antenna’s frequency band. The influence of these critical parameters on the frequency band was examined based on actual simulation conditions. Non-essential parameters that did not significantly impact frequency variations were omitted and will not be discussed. To demonstrate the working effects of the proposed MIMO antenna, variations were introduced in the strip parameters L_1_ and L_2_ for each antenna element. Figure 5 displays the simulated S_11_ parameter as it changed with different values of L_1_, L_2_, L_4_, and L_5_ while keeping other optimized parameters constant. A comparison of various L_1_ and L_2_ values revealed their distinct influence on low- and high-frequency resonances, respectively. As illustrated in Figure 5a, significant changes occurred in the low-frequency band of the antenna when altering the value of L_1_. Specifically, the frequency decreased as the length of the L-shaped strip, L_1_, increased. Conversely, modifying the length of the C-shaped strip line, L_2_, led to notable fluctuations in the high-frequency band of the antenna, as depicted in Figure 5b. It is evident from Figure 5c,d that L_4_ primarily affected the low-frequency band, while L_5_ had a greater impact on the high-frequency band. Overall, compared to the influence of L_1_ and L_2_ on the antenna’s operating frequency, the effect of L_4_ and L_5_ on the frequency band was relatively smaller when changing the same value. This observation is further supported by the vector current distribution diagram shown below. Consequently, the resonant frequency of the proposed antenna element can be independently adjusted, showcasing a unique characteristic that facilitates straightforward operation in engineering applications.

**Figure 6:**
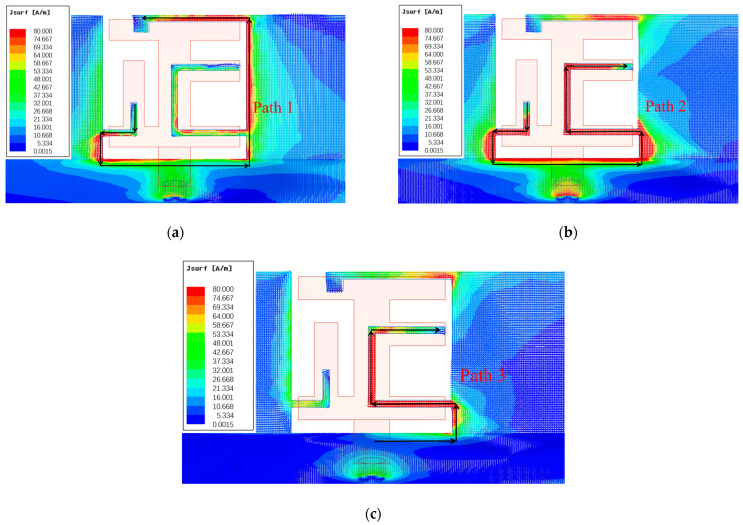
Surface electric field distribution of the antenna at (**a**) 3.5 GHz, (**b**) 4.9 GHz, and (**c**) 5.5 GHz.

To enhance our understanding of the operational principle of the antenna element, we present the surface electric field at 3.5 GHz, 4.9 GHz, and 5.5 GHz in Figure 6. Initially, the surface electric field demonstrated its peak intensity at the two inverted L-shaped strips on the side-edge metal frame, with the current concentrated along the edge of the metal frame at 3.5 GHz. Subsequently, at 4.9 GHz, a robust electric field path was distributed along the bottom of the metal frame and the C-shaped strip. Finally, in the 5.5 GHz band, the maximum electric field values were observed at the top edge of the metal frame and the C-shaped strip. The current vector distribution of the antenna primarily concentrated on path 1 at 3.5 GHz, as depicted in Figure 6a. Moreover, both path 2 and path 3 are illustrated in Figure 6b,c, showcasing the direction of current flow in their respective operating bands. These findings signify that a portion of the metal frame, along with the ground, functions as the radiator of the antenna. Consequently, the resonances in the differential bands are independently associated with the fundamental modes of the distinct current paths.

## 4. Experimental Results and Discussion

### 4.1. S-Parameters

**Figure 7:**
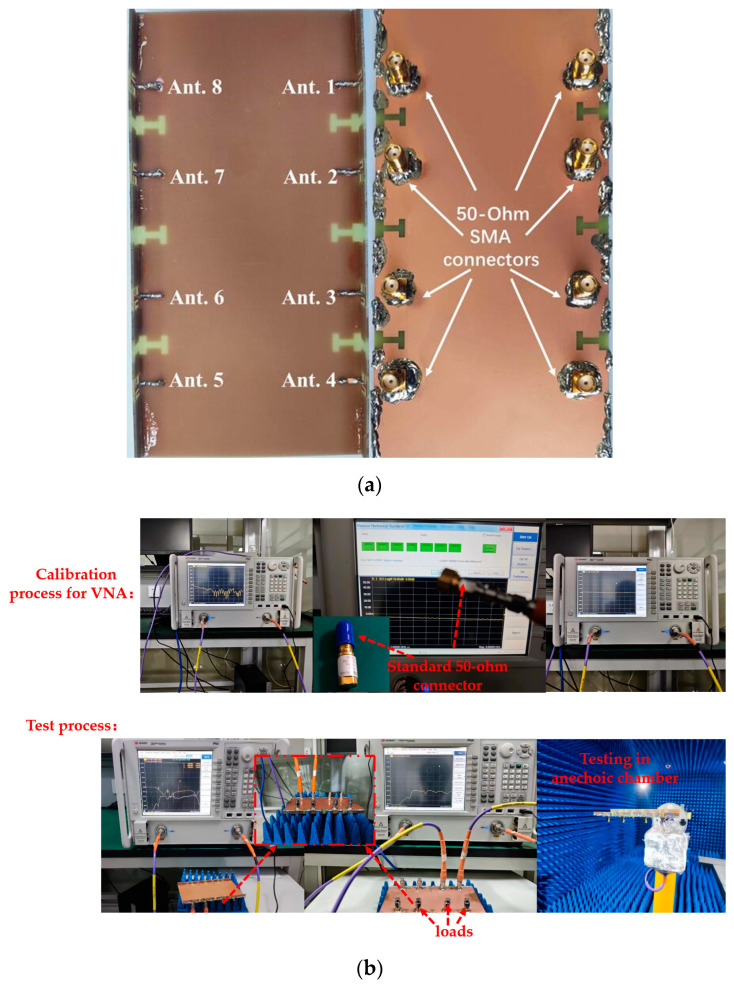
(**a**) Photos of the fabricated prototype: top view and bottom view. (**b**) Photos of the testing process.

**Figure 8:**
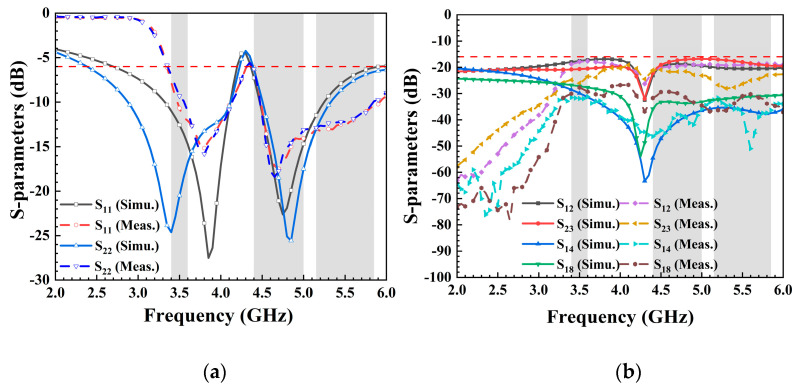
Measured S-parameters: (**a**) reflection coefficients; (**b**) transmission coefficients.

The presented 8-port antenna array was successfully manufactured and subjected to measurements, with the top and bottom views showcased in Figure 7. Each element was connected to a 50-Ohm SMA connector, and the S-parameters were obtained using a Vector Network Analyzer (VNA) with model number N5224A produced by Keysight Technologies (Santa Rosa, CA, USA). The essential parameters of MIMO antennas, such as radiation pattern and efficiency, were evaluated in a standard microwave anechoic chamber. The detailed testing procedure is depicted in Figure 7b. Firstly, the calibration of the vector network analysis test port was performed, demonstrating the calibration process utilizing a standard 50-Ohm connector. Secondly, when examining the reflection coefficient and transmission coefficient of ports 1 and 2, all ports were connected to other standard matched loads, thus restoring the authentic operation of the antenna to the greatest extent. Finally, the specific process for measuring the radiation pattern, antenna efficiency, and other parameters of the antenna in the standard microwave anechoic chamber is presented. Figure 8 showcases the remarkable agreement between simulation and measurement results. In Figure 8a, it is observed that the measured S_11_ was below −6 dB within the designated frequency band (3.36–4.2 GHz and 4.37–5.95 GHz), while the measured transmission coefficients (S_12_, S_23_, S_14_, and S_18_) all fell below −16.5 dB within the desired frequency range. The −6 dB bandwidth met the standards for a mobile phone antenna operating in sub-6 GHz bands. The isolations between nonadjacent antennas significantly surpassed those between adjacent antennas, and while not depicted in the figure, they were well within acceptable ranges. The measured S-parameters affirm that the proposed 8-port MIMO antenna effectively covered the 5G China band N78 (3.4–3.6 GHz), N79 (4.4–5.0 GHz), and WiFi 5 (5.15–5.85 GHz). The disparities between simulated and measured outcomes could arise from differences in the SMA connector used for simulation compared to measurement, as well as welding factors and operational errors.

### 4.2. Radiation Performance

**Figure 9:**
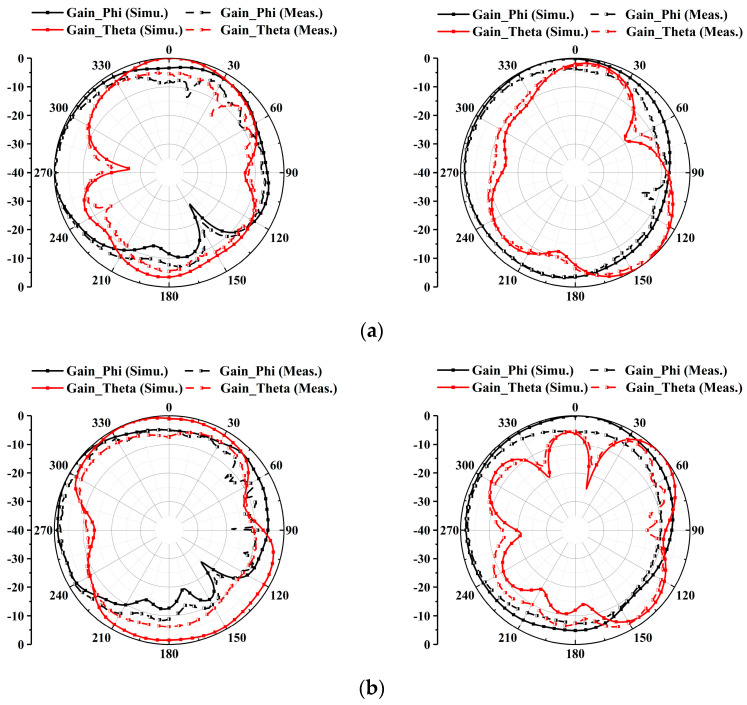

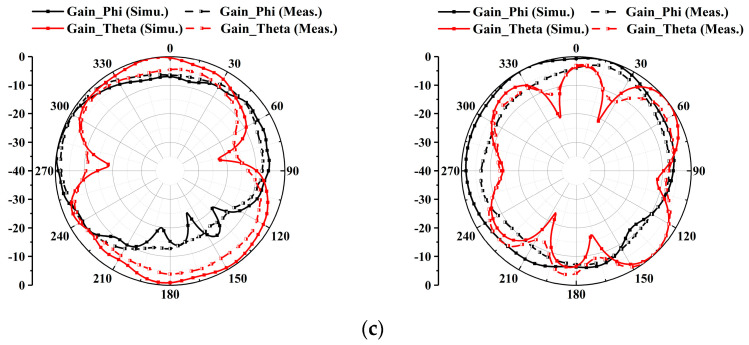
Simulated and measured radiation patterns in xoy-plane and yoz-plane at (**a**) 3.5 GHz, (**b**) 4.9 GHz, and (**c**) 5.5 GHz.

To validate the radiation performances, the proposed antenna underwent measurements in a microwave anechoic chamber. Figure 9 illustrates the simulated and measured theta-polarized and phi-polarized radiation patterns of the antenna at 3.5 GHz, 4.9 GHz, and 5.5 GHz. The close alignment between simulated and measured results indicates favorable radiation characteristics at the three representative frequencies (3.4–3.6 GHz, 4.4–5.0 GHz, and 5.15–5.85 GHz), highlighting promising radiation performance for mobile communications.

### 4.3. MIMO Performance

**Figure 10:**
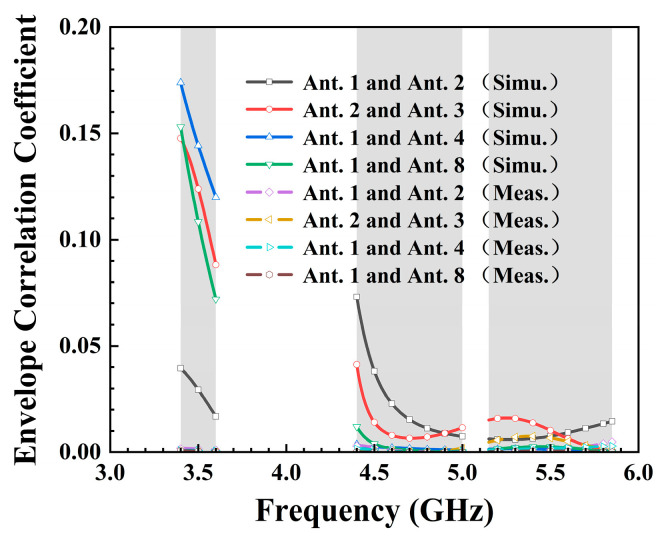
Simulated and measured envelope correlation coefficient.

An essential metric for evaluating MIMO antennas, the envelope correlation coefficient (ECC), was calculated using Equation (1). It signifies the correlation among the received signal amplitudes across various antenna units, serving as a metric to gauge the diversity and coupling performance of MIMO multi-antenna systems. In the operating 5G China band N78 (3.4–3.6 GHz), the maximum ECC was 0.18, occurring between Ant. 1 and Ant. 4. For 5G NR N79 (4.4–5.0 GHz), the ECC between adjacent antenna elements remained below 0.08, as illustrated in Figure 10. Additionally, all simulated ECCs for WiFi 5 (5.15–5.85 GHz) did not exceed 0.03. Based on the ECC test results, it was observed that the tested ECC exhibited lower values in the three target frequency bands when compared to the simulation results. The consistently low ECC values affirm the proposed 8-port antenna’s exemplary diversity performance for a 5G MIMO system.

(1) $$\rho_{c}(i,j)=\frac{{|}_{4\pi }^{\iint }[F_{i}(\theta ,\varphi )\cdot F_{j}^{*}(\theta ,\varphi )]d\Omega {|}^{2}}{{\;}_{4\pi }^{\iint }|F_{i}(\theta ,\varphi ){|}^{2}d\Omega \cdot {\;}_{4\pi }^{\iint }|F_{j}(\theta ,\varphi ){|}^{2}d\Omega }$$

### 4.4. Total Efficiency

**Figure 11:**
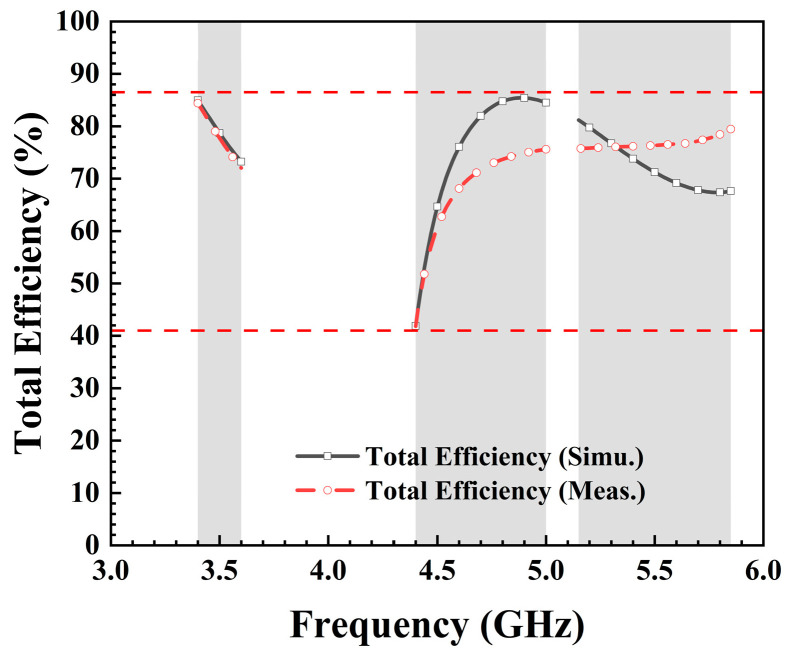
Simulated and measured total efficiency.

The overall efficiency of an antenna typically indicates its capability to convert input power into radiated power, directly impacting the transmission range and reliability of the wireless communication system. Higher antenna efficiency results in more effective conversion of transmitted power into electromagnetic waves, thereby enhancing signal propagation range and coverage area. The measurement of an antenna is typically categorized into active and passive testing. In active testing, the emphasis lies on assessing factors such as the antenna’s radiation pattern, polarization, and impedance characteristics as it operates in real time. Passive testing is primarily utilized to assess the radiation performance of the antenna, and it focuses on measuring parameters such as antenna efficiency, gain, and radiating pattern. The process of measuring antenna efficiency involves several steps. The proposed antenna was positioned in a microwave anechoic chamber, to minimize external interference. A signal was then transmitted to the antenna using a vector network analyzer. The received power and the output power of the signal source were meticulously measured and compared. Antenna efficiency was subsequently calculated based on these measurements. It was crucial to maintain a reliable connection between the signal source and the antenna to minimize signal loss and ensure precise measurements. Additionally, the process required equipment calibration and careful consideration of potential sources of error. Overall, measuring antenna efficiency demands precision and meticulous attention to detail. As depicted in Figure 11, the total efficiency of both simulated and tested antennas ranged from 41.5% to 86%. Notably, the total efficiency measured during testing was lower than that of the simulated antenna, possibly attributed to errors in the microwave anechoic chamber and human operational inaccuracies.

### 4.5. Effects of Hand

**Figure 12:**
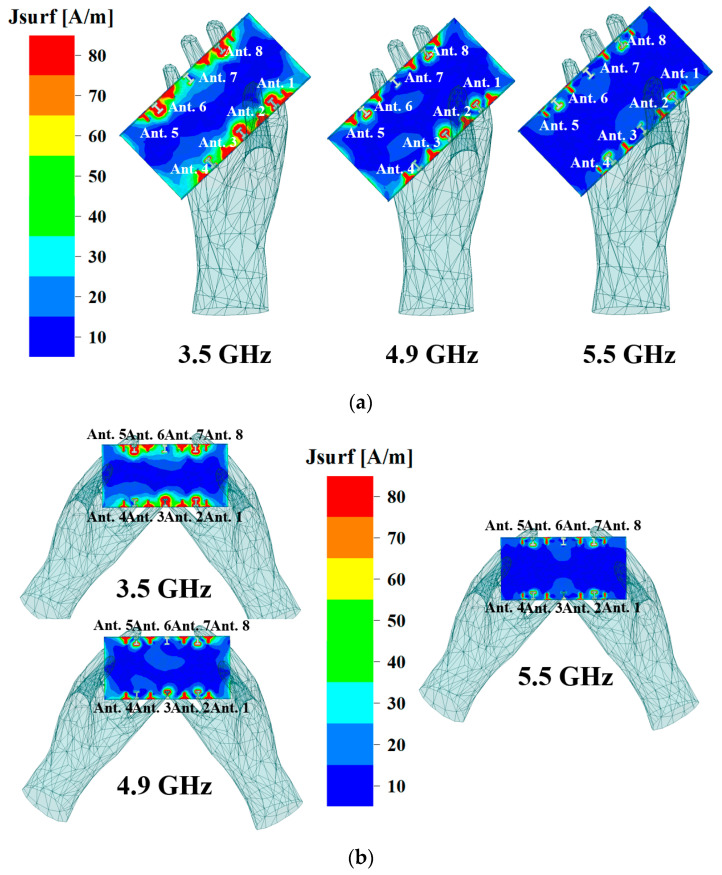
(**a**) The current distribution of the single-hand mode in different bands. (**b**) The current distribution of the double-hands mode in different bands.

**Figure 13:**
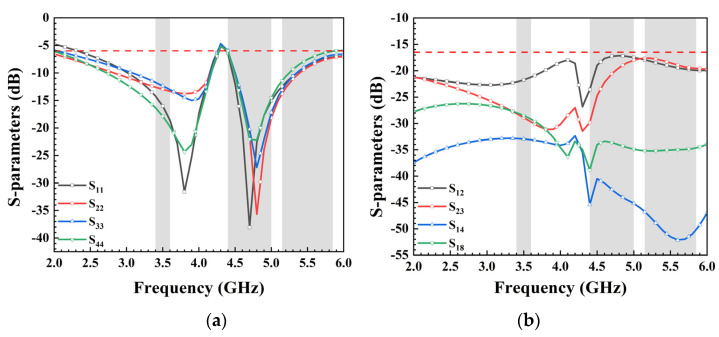

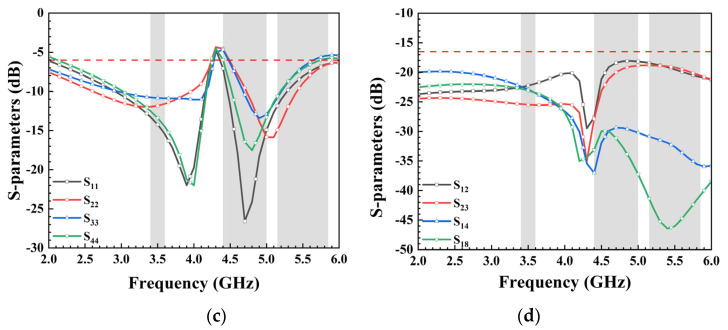
(**a**) The simulated reflection coefficients of the single-hand mode. (**b**) The simulated transmission coefficients of the single-hand mode. (**c**) The simulated transmission coefficient of the double-hands mode. (**d**) The simulated transmission coefficients of the single-hand mode.

When designing wireless electronic products like mobile phone antennas and wearable device antennas for signal transmission and reception, it is crucial to not only assess antenna performance within the device’s physical structure but also account for the influence of the human body on antenna performance. Ansys HFSS 15.0, an electromagnetic simulation software, offers a human body structure model. Within this model, material properties such as relative permittivity and bulk conductivity are predefined as global variables, specifically denoting the average values of relative permittivity and volume conductivity. Considering the impact of human body tissues on antenna performance due to their lossy properties and high permittivity, Figure 12a,b depict the effects of hand phantoms on the current distribution of the proposed MIMO antenna under single-hand mode and double-hands mode. Simulated S-parameters of the user’s hand are presented in Figure 13. For the single-hand mode (Figure 13a), the hand’s close proximity to Ant. 2 and Ant. 3 led to decreases in the simulated reflection coefficient for these antennas. In the double-hands mode (Figure 13c), Ant. 2, Ant. 3, Ant. 6, and Ant. 7 performance deteriorated because they were mostly covered by fingers. Notably, Figure 13b,d demonstrate that all isolations between different antenna elements remained higher than 16.5 dB. The remaining mobile phone antenna units, unaffected by finger coverage, effectively covered the desired frequency bands while also maintaining satisfactory isolation between each port. These results affirm that the antenna maintained good performance whether held by one or two hands.

Table 1 provides a comprehensive comparison between the current design and previously reported smartphone MIMO antennas. Notably, the proposed antenna exceled in fulfilling the requirements of dual-band MIMO operations, showcasing commendable performance in terms of isolation, efficiency, and ECC. The majority of the referenced works in Table 1 focus on single-mode antennas covering either one or two sub-6 GHz bands, whereas the proposed antenna extends its coverage to three sub-6 GHz bands. Furthermore, when compared to the most referenced antennas integrated into metal-frame smartphones, the proposed antenna exhibited superior isolation, higher efficiency, and a broader bandwidth. Despite the ECCs in the 3.5 GHz frequency band being slightly higher than those found in the references, the ECC values of the proposed antenna still met the operational demands of the MIMO system.

## 5. Conclusions

In conclusion, a dual-band, 8-port MIMO antenna for 5G/WiFi 5 smartphones was successfully designed, fabricated, and tested in this paper. The antenna exhibited favorable performance, with acceptable ECCs for the MIMO system within the operating bands (3.4–3.6 GHz, 4.4–5.0 GHz, and 5.15–5.85 GHz) and isolation exceeding 16.8 dB. The antenna efficiencies surpassed 41.5% in the target frequency bands. The calculated ECCs from measured electric field results were below 0.18 in the operating bands. Overall, the proposed MIMO antenna array demonstrated significant promise for 5G massive MIMO mobile communication systems.

## Figures and Tables

**Table 1:** Comparison between the proposed 5G MIMO antenna and other relevant designs.

Reference	Bandwidth (GHz)	Isolation (dB)	ECC	Total Efficiency (%)	MIMO Order	Metal Frame
[1]	3.3–4.2, 4.4–5.0, 5.15–5.925 (−6 dB)	>11	<0.1	40–71	8	yes
[4]	3.4–3.6, 4.8–5.1 (−6 dB)	>10	<0.08	41–72, 40–85	8	yes
[5]	3.3–4.2, 4.8–5.0 (−6 dB)	>10	<0.12	53.8–76.5, 40–85	8	yes
[13]	3.3–6 (−10 dB)	>18	<0.08	69.85–90	8	yes
[20]	3.3–3.84, 4.61–4.91 (−10 dB)	>15	<0.02	76–85, 66–82	4	no
[21]	3.4–3.8, 4.8–5.0 (−6 dB)	>15.5	<0.07	42–83, 40–85	8	no
[22]	3.4–3.8, 5.15–5.925 (−6 dB)	>11	<0.1	52.4–71.7, 48.9–75.4	10	no
Proposed work	3.4–3.6, 4.4–5.0, 5.15–5.85 (−6 dB)	>16.5	<0.18	72–82.4, 41.5–75.6	8	yes

## Data Availability

Data are contained within the article.
